# Targeting Microglial K_**ATP**_ Channels to Treat Neurodegenerative Diseases: A Mitochondrial Issue

**DOI:** 10.1155/2013/194546

**Published:** 2013-06-16

**Authors:** Manuel J. Rodríguez, Margot Martínez-Moreno, Francisco J. Ortega, Nicole Mahy

**Affiliations:** ^1^Unitat de Bioquímica i Biologia Molecular, Facultat de Medicina, Institut d'Investigacions Biomèdiques August Pi i Sunyer (IDIBAPS), Universitat de Barcelona and Centro de Investigación Biomédica en Red sobre Enfermedades Neurodegenerativas (CIBERNED), UB c/Casanova 143, E 08036 Barcelona, Spain; ^2^Neurometabolic Disease Lab, Hospital Duran i Reynals, L'Hospitalet de Llobregat, E 08907 Barcelona, Spain

## Abstract

Neurodegeneration is a complex process involving different cell types and neurotransmitters. A common characteristic of neurodegenerative disorders is the occurrence of a neuroinflammatory reaction in which cellular processes involving glial cells, mainly microglia and astrocytes, are activated in response to neuronal death. Microglia do not constitute a unique cell population but rather present a range of phenotypes closely related to the evolution of neurodegeneration. In a dynamic equilibrium with the lesion microenvironment, microglia phenotypes cover from a proinflammatory activation state to a neurotrophic one directly involved in cell repair and extracellular matrix remodeling. At each moment, the microglial phenotype is likely to depend on the diversity of signals from the environment and of its response capacity. As a consequence, microglia present a high energy demand, for which the mitochondria activity determines the microglia participation in the neurodegenerative process. As such, modulation of microglia activity by controlling microglia mitochondrial activity constitutes an innovative approach to interfere in the neurodegenerative process. In this review, we discuss the mitochondrial K_ATP_ channel as a new target to control microglia activity, avoid its toxic phenotype, and facilitate a positive disease outcome.

## 1. An Imbalanced Four-Partite Synapse Crosstalk

The diversity of clinical phenotypes of neurodegenerative diseases share common neuropathological features that underlie significant modifications of the physiological glia-neuronal crosstalk. Over time, this leads to neuronal damage, specific pathway dysfunctions, and neurological disability [1]. Initially, the four-partite synapse, that is, the presynaptic and postsynaptic neurons, astrocytes, and microglia, presents a diversity of new communications that render and maintain a chronic neuroinflammation [1–3] with activation of various well-coordinated adaptive mechanisms to avoid or reverse damage [1, 3]. In fact, this imbalanced four-partite synapse crosstalk underlies the disease pathogenesis, and its progression—and also brain aging—reflects at each moment the contributions of each cell type. Because of that, higher perturbations are associated with significant molecular and cellular changes that lead to a progressive chronic neurodegenerative process from which return to physiological conditions is impossible [4]. As such, the disease progression represents the dynamic communicative process between all the synaptic participants in which the equilibrium between damaging and neuroprotective signals favors permanently and increasingly neuronal damage and synaptic loss.

The pivotal role of these four cell types in disease has yielded a huge amount of information to explain the dysfunction or disruption of neural circuits. At the present time, the working theories attribute key roles to protein misfolding, excitotoxicity, and mitochondrial dysfunction, this last one related to integrity, bioenergetics, calcium homeostasis, or reactive oxygen species (ROS) generation. In fact, all these elements are interdependent, and all theories consider the participation of the same four-partite synapse organization, in which microglia, as a sensor and effector of CNS immune function, directly influence the disease initiation, progression, and outcome.

## 2. Microglia and Neurodegeneration

### 2.1. Misfolded Protein Diseases

Neurodegenerative diseases can be classified as misfolded protein diseases (MPD) because each one presents specific misfolded proteins. These proteins share no common sequence nor common structural identity between them and include the Protease-resistant Prion Protein, Polyglutamine, Amyloid-*β*, Tau protein, *α*-synuclein, superoxide dismutase 1 (SOD1), TAR DNA-binding protein 43 (TDP-43), or fused in Sarcoma (FUS) [5, 6]. These misfolded proteins reveal a failure to adopt a proper protein folding due to an enhanced production of abnormal proteins or to a perturbation of cellular function and aging under the effects of ROS [7, 8]. In addition, their insufficient clearance also reflects a chronic impaired microglia autophagy resulting in accumulation of protein aggregates, cell damage, and progressive death [1, 9]. For example, in amyotrophic lateral sclerosis (ALS), the misfolded proteins like SOD1 released initially from motor neurons activate microglia, and the ensuing neuronal injury depends upon a well-orchestrated dialogue between motor neurons and microglia [10]. Phagocytic microglial cells are very efficient scavengers but with a limited capacity towards *α*-synuclein aggregates [11, 12] characteristics for Lewy body disorders (LBDs), in particular for Parkinson's disease. The same microglia phagocytosis triggers the release of proinflammatory cytokines, chemokines, and ROS, which may further promote neuronal dysfunction and degeneration and misfolded protein overload [13, 14]. In addition, microglia may also participate in the LBDs pathophysiology due to variations of their human leukocyte antigen region [15]. Thus, in all the different situations, the protein aggregates initiate and maintain a chronic activation of microglia as a constitutive element of MPD with their subsequent direct participation in neural damage through chronic ROS formation and cytokine secretion [16–18].

### 2.2. Excitotoxicity

Regarding excitotoxicity, the alterations of synaptic glutamate and calcium homeostasis lead to neuronal and glia glutamate receptor overactivation, culminating in neuronal death and progressive neural circuit dysfunctions [19, 20]. In this situation, three main compensatory responses are activated to avoid glutamate-induced damage [21]. Thus, the direct activation of the retaliatory systems, based on a coordinated increase of GABA, taurine, and adenosine signaling, will reduce glutamate receptor activation [22, 23], and the rapid adaptation of calcium homeostasis with the neuronal and astroglial formation of calcium precipitates will reduce the increased calcium signaling associated with glutamate [3, 24], and the reduction of glutaminase activity of the astrocyte-neuron glutamate/glutamine cycle will reduce glutamate level in the presynaptic neuron and synaptic glutamate activation [25]. Microglial cells are the glial cell type least susceptible to excitotoxicity, because they mostly express glutamate receptors when they are reactive. Injured neurons would directly activate microglia [26] by the release of a diversity of factors like galectin-3, [27], cystatin C, chemokine (C- X3-C motif) ligand 1 (CX3CL1) [28], or the danger-associated molecular pattern (DAMP) ligands that bind to Toll-like receptors (TLR) [29]. The benefits of such inflammation may be substantial to help avoid further neuronal damage if exposure to excitotoxicity is limited in time or intensity [25, 30]. Otherwhise, microglia effects will be deleterious through chronic ROS formation and cytokines secretion and directly related to the neurodegenerative process. With aging, this process will be potentiated by the senescent affectation of microglia, which changes cell morphology and functions [31–33]. In fact, cumulative evidence indicates a direct pathogenic role of senescent microglia in degenerative CNS diseases [34–36].

### 2.3. Energy Demand and Microglia Reaction

Microglia do not constitute a unique cell population but rather, present a range of phenotypes [1, 37] closely related to the evolution of the lesion process [33]. In a dynamic equilibrium with the lesion microenvironment, these phenotypes range from the well-known proinflammatory activation state to a neurotrophic one directly involved in cell repair and extracellular matrix remodeling [38], with a diversity of intermediary mixed phenotypes that present or not a phagocytic activity [39]. All these adaptative phenotypes relate directly to the evolution of the lesion, and variations in the expression of toxic, protective factors, and phagocytic activities greatly determine the outcome of the tissue [33, 37, 40].

In addition, microglia-like neurons present a high-energy demand, for which the lack of a mitochondrial DNA (mtDNA) repair system determines mtDNA cumulative defects with aging. As said previously, in these conditions, senescent microglia become increasingly dysfunctional and participates in the direct development of neurodegeneration [40, 41]. As in other cells, calcium signaling governs the communication between cytosol and mitochondria [42]. In macrophages, the phagocytic response represents a burst of ROS formation through an increased activity of the NADPH oxidase and also of mitochondria. The mitochondria ROS (mtROS) are potentiated by the translocated tumor necrosis factor receptor-associated factor 6 (TRAF6) [43] and its interaction with the ubiquitinated protein evolutionarily conserved signaling intermediate in Toll pathways (ECSIT) [44]. ECSIT associates with the oxidative phosphorylation complex I components and facilitates the assembly of the mitochondrial electron transport chain [44]. Thus, in macrophages, TRAF6 translocation induces the juxtaposition of phagosomes and mitochondria and potentiates mtROS formation and energy production [45]. A similar TRAF6 recruitment to mitochondria that engages ESCIT on the mitochondria surface is also observed in the same cells upon lipopolysaccharide (LPS, a TLR4 agonist) treatment [43].

In microglia, recruitment of cytoplasmic TRAF6 modulates LPS-evoked cytokine release [46]. The great similarity between peripheral and central immune systems, in particular between macrophages and microglia, makes a similar action of TRAF6 in microglia activated by LPS possible. If true, the mitochondria respiratory burst rending increased energy and ROS production in response to the elevated calcium required for the adoption of a specific microglia phenotype would also depend on TRAF6 translocation and interaction with ECSIT. And how calcium variations and TRAF6 interact to modulate their microglia effects should help identify the molecular pathways linked to the adoption of a phenotype or another one.

In parallel, a defect in the astrocyte-neuron crosstalk does not ensure sufficient neuronal supply of glucose, lactate, and oxygen, leading to increased neuronal damage, probably impossible to effectively repair [47]. This damage results in subsequent switch in microglia phenotype from an initial neuroprotective one to a final proinflammatory one and to the disease onset and progression [1].

Thus, whether microglia adopt a phenotype that will exacerbate tissue injury or one to promote brain repair and phagocytosis is likely to depend on the diversity of signals from the lesion environment and of the microglia response capacity [48, 49]. At each moment, microglia effectiveness to adapt to the changing synaptic signals and phagocytic needs to rapidly remove cellular debris depends on its accurate adaptation to match its immediate energy demand. This implies that to ensure and maintain an activated stage and adapt constantly their expression and function to a determined phenotype, microglia have to correspond permanently with a balanced ATP availability from aerobic glycolysis [50]. However, like in astrocytes, in reactive microglia, part of glucose renders ATP and lactate through anaerobic glycolysis. Thus, in the postulated four-partite synapse, lactate will be shuttling to neurons not only from astrocytes but also from microglia.

Mitochondrial bioenergetics depends on a proper stimulation of mitochondrial oxidative phosphorylation to produce enough ATP to the cell and on the same mitochondria morphology [41, 51]. This requires a constant dynamic and rapid response of its array of individual mitochondria to adapt their shape and size to the microglia energy demand. Thus, via the combined fusion and fission events that are mediated by large guanosine triphosphatases, they improve their number and optimize their regional networks in order to deliver at each moment the needed ATP [52]. In microglia, a higher number of mitochondria mark the transition from microglia resting state to the activated one, as shown by an increased expression of the mitochondrial peripheral benzodiazepine receptor (PBR) labeled with (R)-PK11195 [53]. Both transmission electron microscopy (TEM) and atomic force microscopy (AFM) procedures evidenced that this mitochondrial PBR complex functions as a pore, allowing the translocation of cholesterol and other molecules through the inner mitochondrial membrane [54]. Presently, the (R)-PK11195 ligand is commonly used to quantify microglia in brain sections from excitotoxic damage like stroke, brain trauma, epilepsy, or chronic neurodegenerative disorders [38, 55, 56] and also for PET detection as an in vivo marker of active disease [57].

The mtDNA is arranged in nucleoprotein complexes evenly distributed along the mitochondrial network [58]. Stimulation of mitochondrial fusion by mitofusin 1 and 2 and OPA1 maximizes oxidative phosphorylation because the fused mitochondria share and homogenize the content of all their compartments. Thus, when the mtDNA mutation load is minor, integration of defective mitochondria through fusion complementation increases their oxidative capacity by sharing RNA components and proteins, because the lack of a functional component in one mitochondria can be complemented by the presence of the component in another one [59]. If the mutation load is major, fission activation by Drp1 and Fis 1 increases the number of mitochondria and maintains their physiological functions removing the defective parts by mitophagy [60, 61]. Otherwise, abnormal mitochondrial network dynamics develop, rendering dysfunctional cells as a direct participant of the degenerative process. Presently, this physiopathological mechanism has not yet been evidenced in microglia. The known diseases associated with defects in mitochondria fusion-fission factors, like the OPA1-linked autosomal dominant optic atrophy, are genetic disorders, and not specific of microglia [62]. However, the blockade of glutamate-induced toxicity by antioxidants combined with inhibitors of glutamate uptake argues for a key role of ROS production in excitotoxicity [63]. Also, the importance of some inflammatory-independent neurodegenerative mechanisms associated with mitochondria dysfunction and oxidative stress in multiple sclerosis reinforces the possible participation of defective microglia function in the disease [64]. Thus, unraveling molecular mechanisms amenable to prevent or reverse defective microglia mitochondrial networks would open new possibilities to control neurodegenerative disorders.

The rise in cytosol calcium signaling associated with activation of glutamate and cytokine receptors of microglia match microglia cell energy demand with mitochondria supply. Upon activation, microglia express glutamine synthase (GS) and the two transporters EAAT-1 and -2 for glutamate and glutamine synaptic extrusion [65]. In mitochondria, glutamate and glutamine render oxoglutarate for the Krebs cycle and ammonium. At high concentration, ammonium contributes to the generation of superoxide, which after reaction with NO forms the highly reactive peroxynitrite [66]. The progressive reduction of neuronal glutaminase activity that parallels the switch from the apoptotic to the necrotic neuronal death [25] underlies a similar displacement of the glutamate/glutamine cycle toward a reduced glutamate formation and a net glutamine output [25]. So, the highest glutamine level present in necrosis renders more ammonium and more ROS production in microglia—adopting a mostly proinflammatory phenotype—damaging the surrounding cells and also their mitochondria network. This will cause microglia dysfunctions that may result in apoptosis [66].

Finally, stimulation of the mitochondrial calcium uniporter (MCU), located in the inner membrane (MIM), promotes an active mitochondria calcium uptake [67]. Calcium moves down the MIM electrochemical gradient and, in the matrix, stimulates the rate limiting enzymes—pyruvate, isocitrate, and oxoglutarate dehydrogenases and also the ATP synthase—to render increased amounts of ATP and also of ROS [67–70]. Thus, whenever the cause of a major energy demand, a defective protein that does not allow a proper coordinated fission and fusion process induces a mitochondria dysfunction. This will cause a reduced cytosol ATP availability, an increased ROS production, major mtDNA dysfunctions, and a calcium dyshomeostasis, all of them resulting in a definitive loss of mitochondria network integrity and cell abnormalities [41, 71]. In view of the abovementioned theories, which all consider the participation of cellular and molecular multidirectional synaptic interactions, identification of therapeutic targets related to disease pathogenesis for the rationale design of treatments remains quite elusive. Modulation of microglia response by controlling their mitochondrial activity constitutes an innovative approach to interfere in the neurodegenerative process. The identification of a mitochondrial K_ATP_ channel expressed in human and rodents is a new target to control microglia activity, avoid their toxic phenotype, and facilitate a positive disease outcome.

## 3. K_ATP_ Channels in the CNS

K_ATP_ channels play important roles in many cellular functions by coupling cell metabolism to electrical activity with the participation of glucokinase. First detected in cardiac myocytes, they have been found in pancreatic *β* cells, skeletal and smooth muscle, neurons, pituitary, and tubular cells of the kidney [72–74]. In these tissues, K_ATP_ channels couple electrical activity to energy metabolism by regulating potassium fluxes across the cell membrane when glucose is available in sufficient conditions [75]. Increased energy metabolism leads to channel closure, membrane depolarization, and electrical activity. Conversely, metabolic inhibition opens K_ATP_ channels and suppresses electrical activity [76, 77].

Plasmalemmal K_ATP_ channels are assembled as a heterooctameric complex [78, 79] from two structurally distinct subunits: the pore forming inwardly rectifying potassium channel (Kir) subunit 6.1 or 6.2 and the regulatory sulphonylurea receptor (SUR). SUR, like all other ATP-Binding Cassette transporters, contains two transmembrane domains and two cytoplasmic ones (Nucleotide Binding Folds 1 and 2). Its N-terminal transmembrane domain mediates SUR-Kir6 interactions [80]. While ATP inhibits the K_ATP_ channel by direct binding to the cytoplasmic Kir6 domains [81], activators like Mg-nucleotides [82] potassium channel openers (KCOs) and inhibitors like sulfonylurea drugs [83] bind SUR to modulate the channel. K_ATP_ channels play a multitude of functional roles in the organism. In endocrine cells, they play an important role in hormone release, including insulin from pancreatic *β* cells [84] and glucagon from pancreatic *α* cells [85]. Epithelial cells of blood vessels also express K_ATP_ channels, where they are involved in the control of blood flow and cerebrovascular processes [86].

In the brain, neuronal expression of K_ATP_ has been described in the substantia nigra, the neocortex, hippocampus, and hypothalamus [72, 74]. In these areas, K_ATP_ channels modulate electrical activity and neurotransmitter release [87], protect against seizures [88], and play an essential role in the control of glucose homeostasis [82]. The expression of K_ATP_ channels has also been suggested in microglia [89]. As discussed in Section 4, our previous studies showed that reactive microglia increase their expression of the K_ATP_-channel components Kir6.1, Kir6.2, SUR1, and SUR2B after brain pathologies such as stroke and Alzheimer's disease (AD) [90–92].

Finally, K_ATP_ channels have also been described in the mitochondria, located on the inner membrane of these organelles where they play a crucial role in the maintenance of mitochondrial homeostasis and of the proton gradient involved in the respiratory chain [93, 94].

### 3.1. K_ATP_ Channel Gating and Pharmacology

The octameric structure of the K_ATP_ channel with four inhibitory ATP-binding sites per channel (one on each Kir6.2 subunit) and eight stimulatory Mg-nucleotide-binding sites on SUR [95] represents a complex regulation by nucleotides. The same for the channel kinetics, with a large number of kinetic states, as its activity reflects at each moment the result of the nucleotide effects at each site. Several endogenous ligands bind the K_ATP_ channel subunits: ATP (with or without Mg^2+^) inhibits the Kir6.2 subunits, and phosphatidylinositol-4,5-bisphosphate activates them; sulphonylureas inhibit the SUR subunits, and KCO drugs activate them. In addition, in the presence of Mg^2+^, ATP and ADP can activate the channel through interaction with the nucleotide-binding folders of SUR [83]. Inhibition by ATP binding to Kir6.2 and activation by Mg-nucleotides is probably the first physiological regulatory mechanism [82].

In recent years, K_ATP_ channels have attracted increasing interest as targets for drug development. Their pivotal role in a plethora of physiological processes has been underscored by recent discoveries linking potassium channel mutations to various diseases. The second generation of KCOs or potassium channel blockers, with an improved in vitro or in vivo selectivity, has broadened the chemical diversity of K_ATP_ channel ligands. However, despite this significant progress, a lot of work remains to be done to fully exploit the pharmacological potential of K_ATP_ channels and their KCOs or potassium channel blocker ligands. Sulfonylureas that bind K_ATP_ channel are oral hypoglycemic agents widely used in the treatment of type II diabetes mellitus [96]. For example, glibenclamide binds with subnanomolar or nanomolar affinity and is a potent inhibitor of SUR1-regulated channel activity [97]. SUR1-regulated channels are exquisitely sensitive to changes in the metabolic state of the cell, responding to physiologically meaningful changes in intracellular ATP concentration by modulating channel open probability [98].

On the other hand, considering the unique role that K_ATP_ channels play in the maintenance of cellular homeostasis, the KCOs family adds its potential in promoting cellular protection under conditions of metabolic stress to the already existing pharmacotherapy. Preclinical evidence indicates a broad therapeutic potential for KCOs in hypertension, cardiac ischemia, asthma, or urinary incontinence to mention a few. For example, diazoxide (7-chloro-3-methyl-4H-1,2,4-benzothiadiazine 1,1-dioxide) is a small molecule well known to activate K_ATP_ channels in the smooth muscle of blood vessels and pancreatic *β* cells by increasing membrane permeability to potassium ions. Diazoxide binds with similar affinities to SUR1 and SUR2B on the interplay between SUR and Kir subunits, a site different of the other KCOs binding sites [83, 99], and its hyperpolarization of cell membranes prevents calcium entry via voltage-gated calcium channels, resulting in vasorelaxation and the inhibition of insulin secretion [100]. For all this, diazoxide has been approved and used since the 1970s for treating malignant hypertension and hypoglycemia in most European countries, the USA, and Canada [101, 102].

## 4. The Mitochondrial K_ATP_ Channel

More than 20 years ago, a putative mitochondrial K_ATP_ (mitoK_ATP_) channel was proposed, both functionally and molecularly distinct from the one in the plasma membrane [103]. Initially, functional mitoK_ATP_ channels were thought to be composed in brain of Kir6.1 or Kir6.2, and SUR2B [104–106] and diazoxide or nicorandil have been proposed as specific mitoK_ATP_ channel openers, whereas 5-hydroxydecanoate would specifically block this channel type [107–111].

Some authors have questioned the existence of the mitoK_ATP_ channel since they failed to detect its existence in isolated heart and brain mitochondria [112, 113], and the specificity of diazoxide and 5-hydroxydecanoate has been questioned [114, 115]. According to these authors, the lack of specific effects of diazoxide could indicate that mitoK_ATP_ channels are not present in mitochondria and that the pharmacological effects of diazoxide and 5-hydroxydecanoate are caused by interaction with other pharmacological or unspecific targets (see [116] for a review). However, one must consider that the variable findings can depend on minor differences in mitochondrial isolation procedures, since the channel may be inactivated during isolation of mitochondria, or a cofactor that needs to be present may be lost [113]. Moreover, the nonspecific effects of diazoxide are observed in vitro when very high concentrations are used (higher than 50 *μ*M). Since in rat heart mitochondria, diazoxide is found to open mitochondrial K_ATP_ channels with a half-maximal saturation of 2.3 *μ*M [94]. These concentrations are in excess compared with those described to promote mitochondrial K_ATP_ channel opening [117, 118].

Putative tissue heterogeneity of mitoK_ATP_ channel expression must also be taken into account to explain the specificity of diazoxide actions. For instance, expression of K_ATP_ channel components was not detected in resting microglia of rat and human brain, whereas activated cells in ischemic rats and AD patients presented strong labeling for SUR and Kir components in the cytoplasm and plasmalemma [90–92]. In this line, diazoxide prevents apoptosis of epithelial cells in the aorta of diabetic rats compared to controls [119]. Thus, in physiological conditions, very low expression of mitoK_ATP_ channels in cardiac tissue or neurons may render cells refractory to low diazoxide concentrations, whereas these tissues may increase the channel expression in pathological conditions.

Within this controversy, a major problem is that the molecular identity of mitoK_ATP_ has not yet been determined. A recent study has presented strong proteomic and pharmacological evidence of kcnj1 as a pore-forming subunit of mitoK_ATP_ channels [120]. This result supports the existence of specific mitoK_ATP_ channels and should help to explain the tissue specificity of different KCOs and clarify why some authors found mitoK_ATP_ channels sensitive to drugs that do not affect the plasmalemmal K_ATP_ one [121].

Because SUR1-regulated channels are exquisitely sensitive to changes in the metabolic state of the cell and that microglia are permanently sensing the environment, the expression of K_ATP_ channels in activated microglia will couple cell energy to membrane potential and microglia response with the adoption of a specific phenotype to the surrounding signals. This channel expression may then be critical in determining, at least in part, microglia participation in the pathogenic process.

### 4.1. MitoK_ATP_ Channels in Neurodegeneration

Our laboratory has described the expression of K_ATP_ channels in microglia [1, 90, 91], which control the release of a diversity of inflammatory mediators, such as nitric oxide (NO), interleukine-6, or TNF-*α* [122]. In a rat model of neurodegeneration and in postmortem samples of patients with AD, we also reported that activated microglia strongly expressed K_ATP_ channel SUR components [90] and that reactive microglia increase their expression of the K_ATP_ channel components Kir6.1, Kir6.2, SUR1, and SUR2B after brain insults [92, 122]. In this context, controlling the extent of microglial activation may offer prospective clinical therapeutic benefits for inflammation-related neurodegenerative disorders. We and other authors have documented that pharmacological activation of K_ATP_ channels can exert neuroprotective and anti-inflammatory effects on the brain against ischemia, trauma, and neurotoxicants [123–126].

Diazoxide has evidenced prevention of rotenone-induced microglial activation and production of TNF-*α* and prostaglandin E2 in cultured BV2 microglia [126]. Also, diazoxide inhibited in vitro NO, TNF-*α*, interleukin-6 production, and inducible nitric oxide synthase expression by LPS-activated microglia. In mitochondria isolated from these cells, diazoxide also alleviated rotenone-induced mitochondrial membrane potential loss [126], for which mitoK_ATP_ channels participate in the regulation of microglial proinflammatory activation.

In vivo studies have confirmed that diazoxide exhibits neuroprotective effects against rotenone, along with the inhibition of microglial activation and neuroinflammation, without affecting cyclooxygenase-2 expression and phagocytosis [126]. When tested in an experimental autoimmune encephalomyelitis (EAE) murine model of multiple sclerosis, 1 mg/kg oral diazoxide ameliorated EAE clinical signs but did not prevent disease. A significant reduction in myelin and axonal loss accompanied by a decrease in glial activation and neuronal damage was observed. In this model, diazoxide did not affect the number of infiltrating lymphocytes positive for CD3 and CD20 in the spinal cord [122]. Finally, diazoxide has been tested in the triple transgenic mouse model of AD (3xTg-AD) that harbors three AD-related genetic loci: human PS1M146V, human APPswe, and human tauP301L [127], and 3xTgAD mice treated with 10 mg/kg/day diazoxide for 8 months exhibited improved performance in the Morris water maze test and decreased accumulation of A*β* oligomers and hyperphosphorylated Tau in the cerebral cortex and hippocampus [128]. In turn, exposition to A*β* oligomers increases the neuronal expression of K_ATP_ channels. With time, this increase is specific for Kir6.1 and SUR2 components [129], which suggest that A*β* oligomers induce differential regulations of K_ATP_ subunit neuronal expression. Exposed to A*β* oligomers, the continuous oxidative stress results in neuronal severe mitochondria dysfunction [130, 131], and the expression changes of K_ATP_ channel components may reflect neuronal attempts to resist the insult in accordance with the metabolic state.

The chronic glutamate-mediated overexcitation of neurons is a newer concept that has linked excitotoxicity to neurodegenerative processes in ALS, Huntington's disease, AD, and Parkinson's disease [19]. Such a chronic excitotoxic process can be triggered by a dysfunction of glutamate synapses, due to an anomaly at the presynaptic, postsynaptic, or astroglial levels [21]. The contribution of excitotoxicity to the neurodegenerative process can be reproduced by the microinjection of low doses of glutamate agonist into the rodent brain. Due to the high affinity of ionotropic glutamate receptors for their specific agonists, such as N-methyl-D-aspartate (NMDA), these drugs injected in non-saturable conditions can trigger calcium-mediated excitotoxicity in several rat brain areas and induce an ongoing neurodegenerative process [55, 56, 132, 133].

For example, any microinjection of NMDA in the rat hippocampus triggers a persistent process that leads to progressive hippocampal atrophy with a widespread neuronal loss and a concomitant neuroinflammation [133]. This paradigm also mimics the alterations of other neurotransmitter systems and neuromodulators found in human neurodegeneration [22].

**Figure 1:**
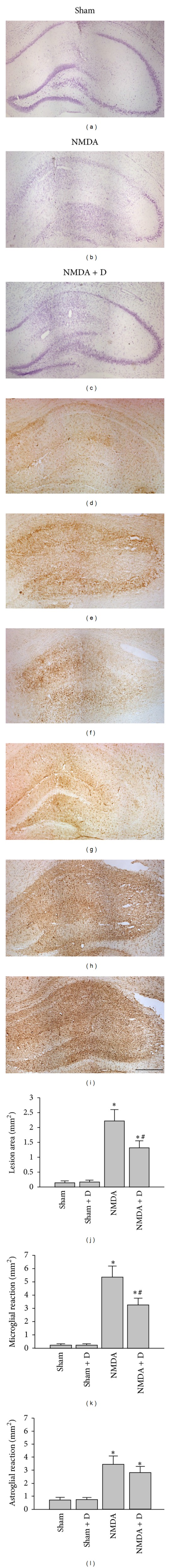
Effect of diazoxide treatment on the NMDA-induced hippocampal lesion. Microphotographs of Nissl-stained sections of a rat hippocampus 15 days after the injection of 0.5 *μ*L of (a) vehicle, (b) 40 mM NMDA, and (c) 40 mM NMDA and treated with 1 mg/kg/day diazoxide p.o. Note that treatment with diazoxide decreased NMDA induced hippocampal lesion. (d) Isolectin B4 histochemistry (IB4) staining of microglia in the hippocampus of sham rats, (e) NMDA-lesioned rats, and (f) NMDA rats treated with diazoxide. Note that treatment with diazoxide decreased the area of enhanced IB4 staining. GFAP immunostaining of the astrocytes in the hippocampus of (g) sham rats, NMDA-lesioned rats (h), and NMDA rats treated with diazoxide (i). Histograms show the quantification of the diazoxide effects in the area of lesion (j), area of microgliosis (k), and area of astrogliosis (l) in the hippocampus of NMDA-lesioned rats. Sham refers to rats injected with vehicle (50 mM PBS, pH 7.4), NMDA refers to rats injected with 0.5 *μ*L of 40 mM NMDA in the hippocampus, and NMDA + D refers to NMDA-injected rats treated with 1 mg/kg/day diazoxide p.o. from postlesion day 5 to 15. Stereotaxic coordinates were −3.3 mm and 2.2 mm from bregma and −2.9 mm from dura [25]. All animals were manipulated in accordance with the European legislation (86/609/EEC), *N* = 6 rats/group. Scale bar 1 mm. *P < 0,05 compared to sham, ^#^ *P* < 0,05 compared to NMDA, LSD (posthoc test).

**Figure 2:**
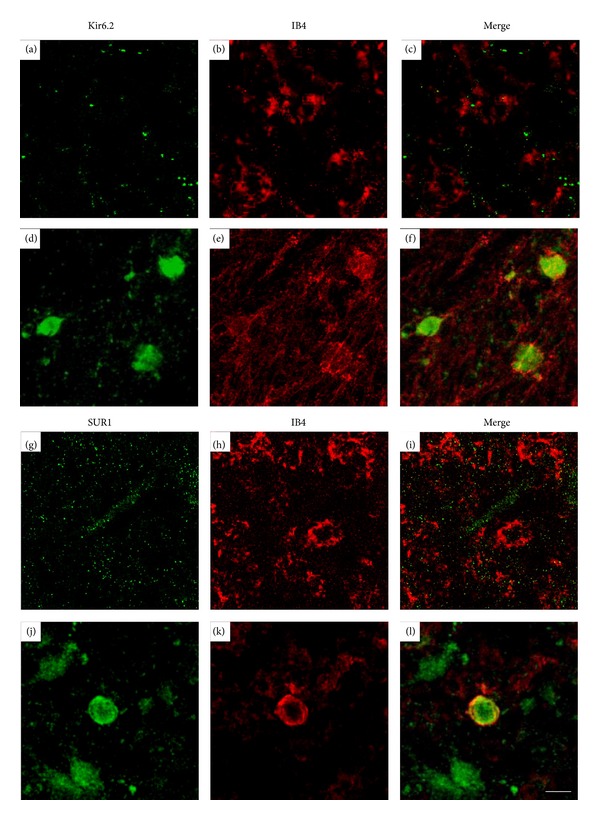
Expression of K_ATP_ channel components SUR1 and Kir6.2 in activated IB4-positive cells into the core of the hippocampal lesion (Bregma −3.3). ((a)–(f)) Confocal photomicrographs of hippocampal sections immunostained with IB4 and anti-Kir6.2 antibodies in control ((a)–(c)) and NMDA-lesioned rats ((d)–(f)). ((g)–(l)) Confocal photomicrographs of hippocampal sections immunostained with IB4 anti-SUR1 antibodies in control ((g)–(i)) and NMDA-lesioned rats ((j)–(l)). Note that reactive amoeboid microglia stained with IB4 show specific immunostaining with anti-Kir6.2 and anti-SUR1 antibodies in the cell membrane but also in the cytoplasm. For lesion details, see legend of Figure 1. Scale bar 10 *μ*m.

This neurodegenerative NMDA-induced hippocampal process is also attenuated by diazoxide oral treatment, which ameliorated microglia-mediated inflammation and reduced neuronal loss (Figure 1). In this model, anti-SUR1 and anti-Kir6.2 antibodies immunostained both the plasmalemma membrane and the perinuclear space of amoeboid reactive microglia (Figure 2), suggesting that microglial activation involves expression and translocation of SUR1 from its internal reservoir toward the cell surface and the mitochondria. Increased expression and translocation of the K_ATP_ channel to the cell surface were also detected by specific binding of fluorescently tagged glibenclamide (glibenclamide BODIPY FL; green fluorescence) to SUR1 in rat primary microglial cultures [92].

Therefore, the expression of mitoK_ATP_ channels by activated microglia indicates that KCOs, such as diazoxide, could be used as therapeutic agents to treat inflammatory processes of neurodegenerative diseases such as multiple sclerosis, Parkinson's disease, or AD.

### 4.2. MitoK_ATP_ Channels Modulate Microglia Reaction

**Figure 3:**
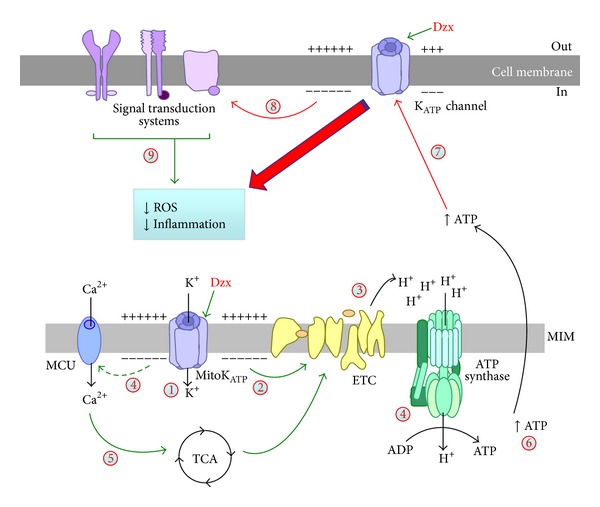
Diazoxide modifies microglial reactivity in the brain. Drawing of the effects of mitochondrial K_ATP_ (mitoK_ATP_) channel opening in the energy metabolism and its consequences in the microglia reaction during neurodegeneration. (1) Diazoxide activates mitoK_ATP_ channels, which (2) depolarizes the mitochondrial internal membrane (MIM), and (3) induces translocation of H^+^ by the electron transport chain (ETC) that enhances both ATP synthesis and activation of the mitochondrial calcium uniporter (MCU) (4). Calcium in the mitochondria activates dehydrogenases of the tricarboxylic acid cycle (TCA) (5) that also enhances ATP production (6). ATP closes the K_ATP_ channel from the plasma membrane (7), while diazoxide opens the channel. As a result, the cell response to activation signals decreases (8), leading to a reduction of the ROS generation and the inflammatory response (9) of reactive microglia (see the text for details).

As explained, depending on the nature of the damage, microglial activation may take place, initially expressing neuroprotective signals to help avoid further neuronal death, but then changing progressively to an inflammatory phenotype [33]. The neuroprotective effects of diazoxide are proposed to modulate the inflammatory microglial activation, without affecting cyclooxygenase-2 expression and phagocytosis [122, 126]. The mechanism of action of diazoxide and other KCOs has not been completely elucidated yet. However, the diazoxide-mediated neuroprotection is supposed to be mediated by its interactions with mitochondria, which are the main ATP-generating sites in microglial cells. In chronically reactive microglia, diazoxide increases potassium flux into the mitochondrial matrix [117, 118] by activation of mitoK_ATP_ channels, which depolarize the MIM and preserve mitochondrial structural and functional integrity [132]. Indeed, cells treated with diazoxide demonstrate a favorable energetic profile with limited damage following stress challenges [108, 134], and mitoK_ATP_ channel opening promotes translocation of H^+^ through the MIM, underscoring the protonophoric uncoupling and enhancing ATP synthesis (Figure 3). As a proposed mechanism, mitoK_ATP_ channels opening serves to maintain constant volume and avoid an excessive mitochondrial contraction that is deleterious for electron transport [117, 118]. Thus, mitoK_ATP_ channel opening may prevent respiratory inhibition due to matrix contraction that would otherwise occur during high rates of ATP synthesis. The increased H^+^ gradient also constitutes the energy source for calcium transport through the MCU towards the mitochondrial matrix [67]. This calcium controls the malate/aspartate transport and dehydrogenases from the tricarboxylic acid cycle, which finally results in increased production of NADH, the electron donor of the respiratory chain. As a result, diazoxide increases the ATP/ADP ratio in the mitochondria and cytoplasm [135] of reactive microglia. ATP closes the K_ATP_ channels from the plasma membrane preventing electrical activity and modifying the cell response to tissue injury. However, specific action of diazoxide on plasmalemma K_ATP_ channel must not be discarded, and the activity of K_ATP_ channel in the membrane will result from a compromise between the ATP-mediated inhibition and the diazoxide-induced opening (Figure 3). As a result, the cell response to tissue injury includes a decreased synthesis of proinflammatory molecules and suppression of mitochondria-derived ROS [126] that result in a maintenance of the mitochondria network integrity and of the phagocytic activity.

Thus, control of mitochondria activity by mitoK_ATP_ channel opening decreases the microglia cytotoxic activity and prevents overactivation of these cells during neurodegeneration. This therapeutic approach may keep the microglia inflammatory activity under the cytotoxic threshold throughout the course of the disease, avoiding amplification of the progressive neuronal loss over time and facilitating a positive disease outcome.
